# Curricula for the teaching of complete dentures in Spanish and Portuguese dental schools

**DOI:** 10.4317/medoral.18078

**Published:** 2012-12-10

**Authors:** Javier Montero, Raquel Castillo-de Oyagüe, Alberto Albaladejo

**Affiliations:** 1 DDS, PhD. Tenured Lecturer in Prosthodontics. Department of Surgery. Faculty of Medicine, University of Salamanca (USAL), Salamanca, Spain; 2 DDS, PhD. Associate Professor, Department of Prosthodontics, School of Dentistry, Complutense University of Madrid (UCM), Madrid, Spain; 3 DDS, PhD. Tenured Lecturer in Orthodontics. Department of Surgery. Faculty of Medicine, University of Salamanca (USAL), Salamanca, Spain

## Abstract

Objectives: Given the need to ensure that dentists are sufficiently skilled to offer the best possible care to their patients, this study aims to evaluate the teaching methods and clinical experience achieved by undergraduate dental students in Spain and Portugal as regards complete dentures. Study design: In February 2011, a questionnaire seeking information about the preclinical and clinical teaching of complete dentures was e-mailed to all Spanish and Portuguese dental schools with fully developed undergraduate degree dental programs. Results: A response rate of 82.6% was obtained. The distribution of lectures and hours spent at the laboratory and in clinical activities revealed that teaching complete dentures is eminently a practical issue, this being mostly performed by full-time prosthodontists. All surveyed schools teach the design of the record base, and most of them instruct students in the mounting of teeth in wax. Most schools (94.7%) used a semiadjustable articulator, alginate for primary impressions (73.7%) and elastomeric materials in border-molded custom trays for final impressions (68.4%). In most schools, within the clinical setting students work in pairs, the mean student/ professional staff member ratio being 2.3 ± 0.7. Most schools perform a competence-based assessment (83.3%), although innovative techniques such as problem-based learning are still rarely applied. On average, the students emplaced 1.8 ± 1.2 complete dentures during their clinical training, ranging from 0 to 4, although no clear trend was seen as regards the minimum number of dentures to be made for graduating. Conclusions: Variations in teaching programs and clinical experience concerning complete denture curricula among Spanish and Portuguese dental schools are evident, but all the schools base their teaching mainly on preclinical and clinical practice. However, the low number of dentures made by student per year seems insufficient to ensure clinical skills and cope with social needs.

** Key words:**Dental education, questionnaires, complete dentures, curricula.

## Introduction

In 1970 Sharry (1) reported that: “there is some agitation and considerable concern over the fact that prosthodontics in dental school curricula is diminishing somewhat in its importance, and that a few dental school administrators believe that complete and partial edentulousness will disappear from the scene within the passage of a decade or two.” However, the need for prosthodontic care could increase for decades in developed countries due to the increasing life expectancy among the elderly and because current denture-wearers require periodic check-ups and replacements. This means that knowledge and skills in treating patients with edentulism will become increasingly important as the century progresses (2).

Clark (3) suggested that edentulous patients could be divided into two main groups: those who can cope with dentures and those who have some difficulty. He also stated that “the undergraduate curriculum should aim to equip graduates to treat the first group properly and attempt to recognise the second group and refer them for specialist care”. Thus, dental schools must continually evaluate the curriculum as regards the construction of removable dentures in order to ensure that the dental health needs of society at large are being met.

It was also predicted that the number of students in dental schools would decline as the 21st century progressed. However, in Spain the number of dentists is in fact growing exponentially (4).

It should be noted that operative dentistry and prosthodontics continue to be the two largest areas in dentistry (5), even in the current graduate curricula in dentistry within the European Higher Education Context. Despite this, since the sixties there has been a gradual reduction in the curricular time devoted to the teaching of both the clinical and technological aspects of complete denture (henceforth CD) construction (5). To a certain extent these changes could reflect changes in population trends and treatment requirements.

In light of the need to ensure that dentists will be sufficiently skilled to offer the best possible care to their patients and for monitoring the baseline clinical competences among European countries according to the recent mandatory implantation of the Bologna Accord for Convergence in Higher Education in the European Union (settled for the academic year 2010-2011), the present study aims to evaluate the current situation regarding teaching methods for CD construction in Spain and Portugal and to determine which educational techniques and materials are currently used, before the implantation of the Bologna System. To date only U.S (6) and British Dental Schools (7) have been evaluated in terms of CD curricula, and there is a clear need of monitoring dental curricula within European countries for convergence reasons. The purpose of this study was to investigate the current situation regarding the teaching of CD construction in Spain and Portugal and to determine which educational techniques and materials are currently in use at the various dental schools (before the implantation of the Bologna System).

## Material and Methods

In February 2011, a questionnaire was sent by e-mail to the chairperson identified as being responsible for the course in CD construction at each of the 23 dental Schools in Spain and Portugal. The questionnaire was designed to capture information concerning the curricular content of the teaching of CD for undergraduates before the implantation of the Bologna System. Following a second mailing to schools that had not replied to the initial attempt within two weeks, 19 of the 23 dental Schools (16 Spanish schools: 11 public and 5 private; and 7 Portuguese: 3 public and 4 private schools) responded, affording a response rate of 82.6%.

The questionnaire consisted of 22 items, and asked the respondents to specify several teaching aspects of their CD programs. Some questions were open, to acquire both quantitative and nominal data with freedom, while others were closed in either a dichotomous or in a multiple-choice format. However, even within these items, the option of providing a specific answer other than the choices listed was also available for some questions. The questions (Q) were pilot-tested by members of dentistry schools of both countries with experience in this field, who approved the questionnaire before it was mailed.

Data were imported to SPSS v.18 for Windows (SPSS Inc, Chicago, IL), and descriptive statistical procedures were carried out to summarize the information.

## Results

All schools still include CD teaching in their curricula, but there are considerable variations in the experience actually gained by students. The responses distribution to the questionnaire is summarized in  Table 1,  Table 2,  Table 3.

**Table 1:**
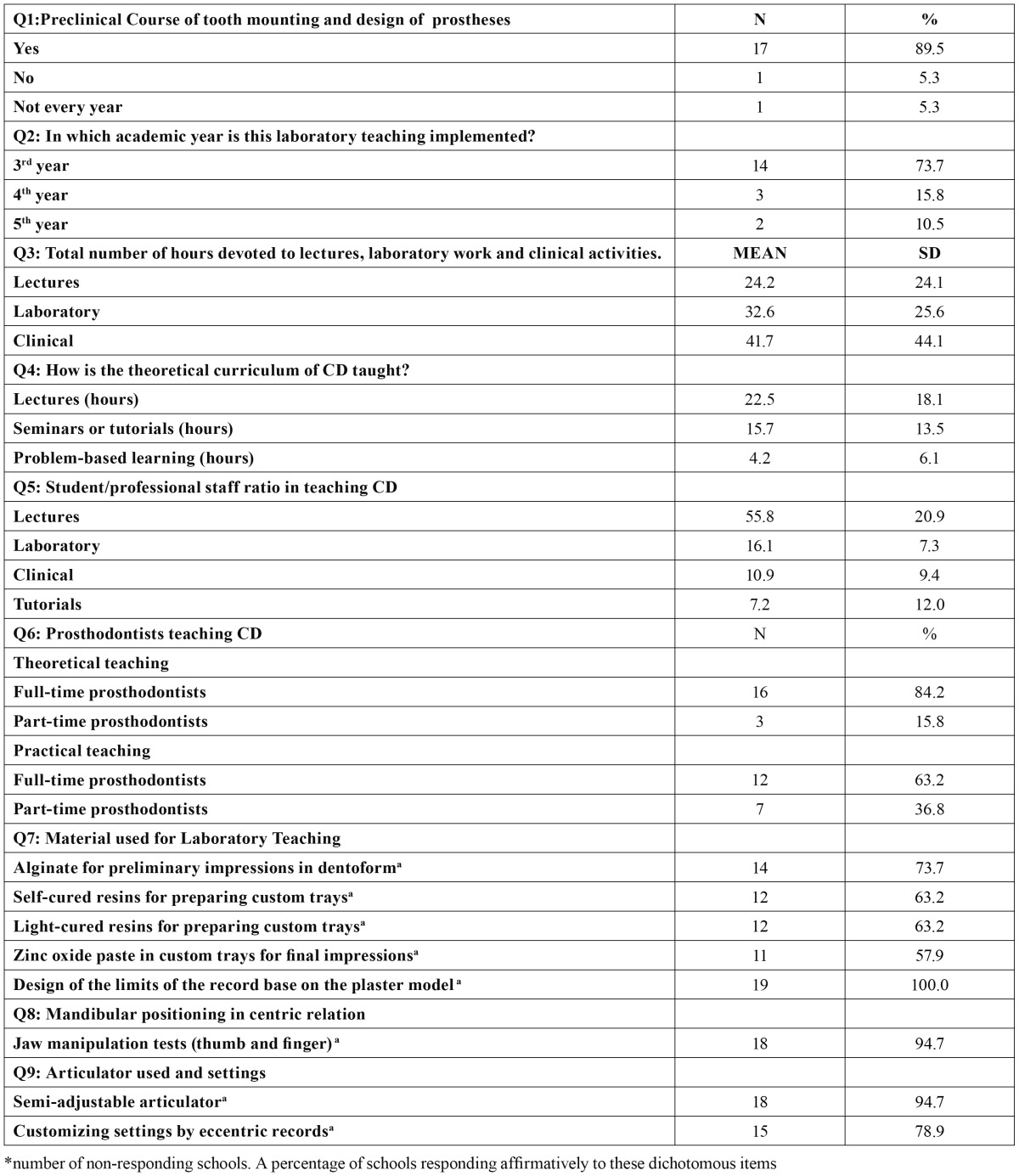
Description of the CD curricula among the Spanish and Portuguese schools surveyed (n=19).

**Table 2:**
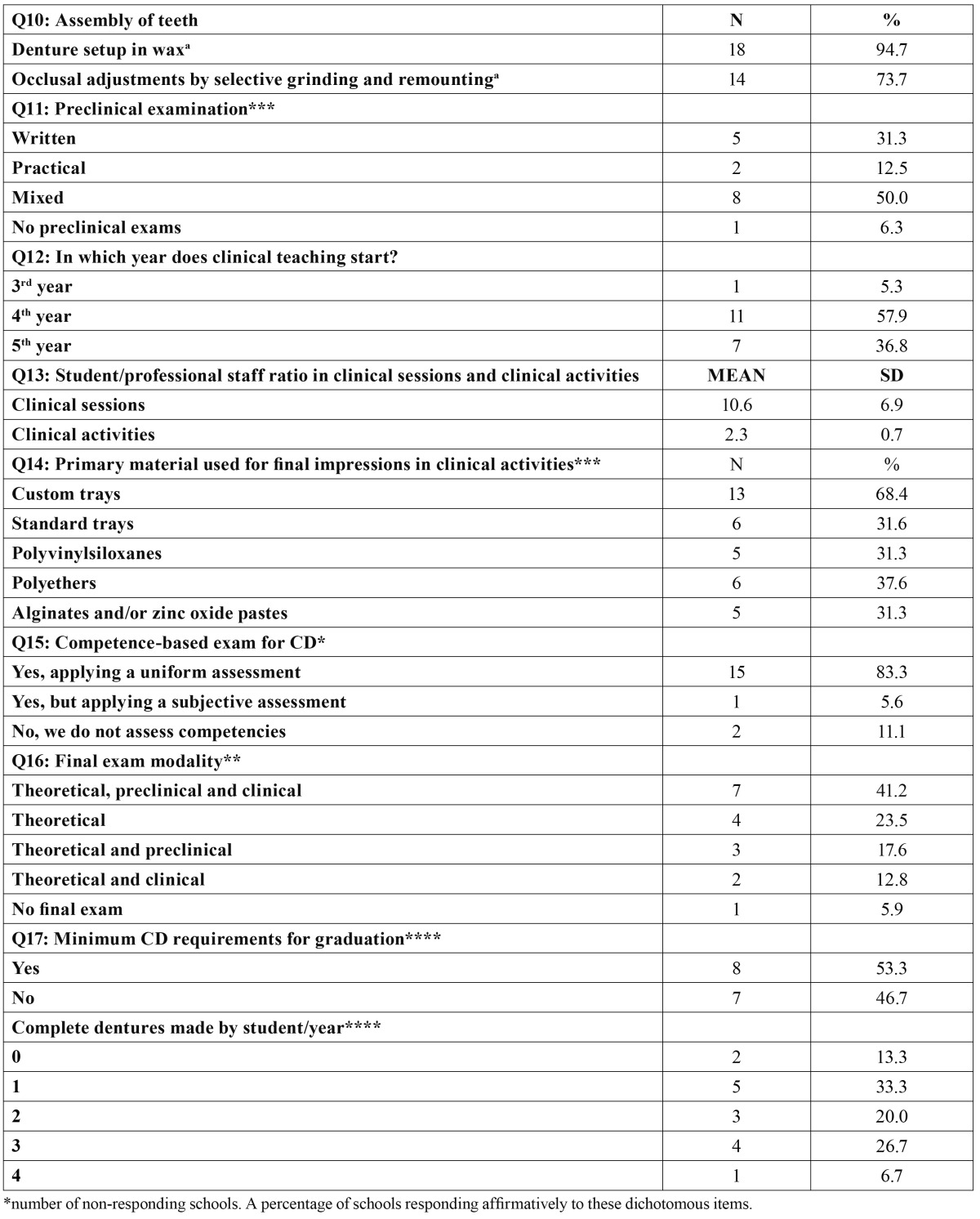
Continuation of the description of CD curricula among the Spanish and Portuguese schools surveyed (n=19).

**Table 3:**
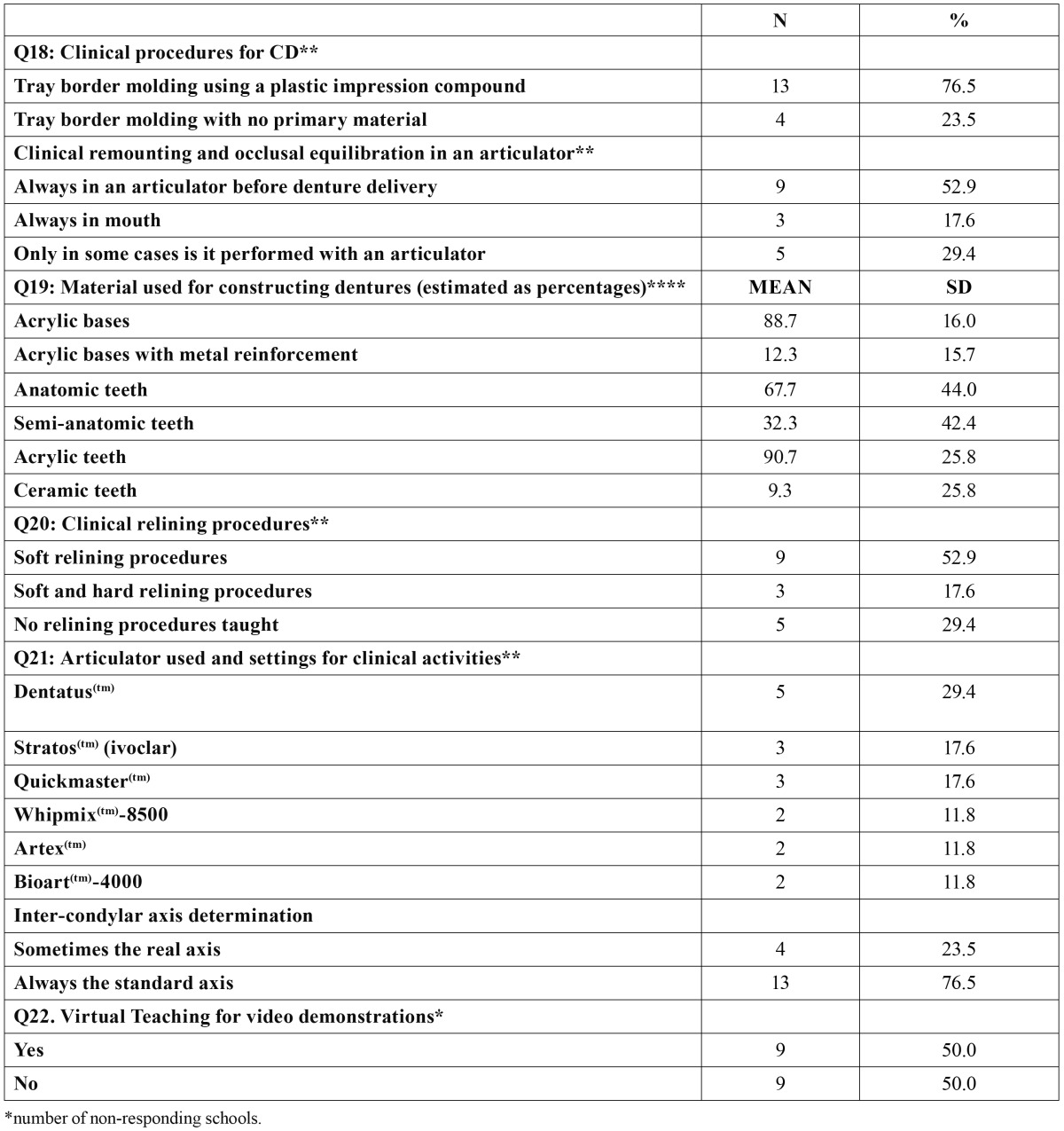
Continuation of the description of CD curricula among the Spanish and Portuguese schools surveyed (n=19).

-Preclinical teaching

Q1: Preclinical course of tooth mounting and the design of prostheses.

Seventeen schools (89.5%) reported that they systematically performed this kind of laboratory teaching in their CD curricula. One school reported that its teachers taught it occasionally (5.3%) and another school declared that it never did so (5.3%).

Q2: In which academic year is your laboratory teaching implemented?

Most schools offer preclinical teaching during the 3rd year (73.7%), but also during 4th year (15.8%) and even during the 5th year (10.5%), which is the last year for Spanish graduates and the penultimate year for Portuguese students.

Q3: Total number of lectures, and hours spent at the laboratory and in clinical activities.

The mean number of hours devoted to lectures was 24.2 ± 24.1, ranging from 2 to 110. The mean number of laboratory hours was 32.6 ± 25.6, ranging from 4 to 110 hours. The mean number of hours spent in clinical activities was 41.7 ± 44.1, ranging from 0 to 120 hours.

Q4: How is the theoretical curriculum for CD taught?

The mean number of hours of formal lectures (one professor teaching a class) was 22.5 ± 18.1, ranging from 5 to 80 hours. The mean number of hours devoted to tutorials and seminars was 15.7 ± 13.5, ranging from 1 to 45 hours. Other techniques, such as problem-based learning and e-learning were also used, with a mean number of hours of 4.2 ± 6.1 ranging from 0 to 15 hours.

Q5: Student-to-professional staff member ratio in teaching CD.

Eight schools (42.1%) reported a preclinical ratio of 10 or fewer students per staff member, and ten schools (52.6%) reported a ratio between 17 and 28 students per staff member. The mean ratio was 16.1 ± 7.3, in a range from 6 to 28 students. The student- staff member ratio for lectures was on average 55.8 ± 20.9 students, ranging between 25 and 100 students. For tutorials, the mean ratio was 7.2 ± 12.0 from a minimum of 1 to a maximum of 44 students. For clinical practice, the mean ratio was 10.9 ± 9.4, in the range of 5 to 40 students per staff member. Thus, the ratio decreases from lectures to tutorials.

Q6: Prosthodontists teaching CD.

Sixteen schools (84.2%) reported that the theoretical teaching of CD was implemented by full-time prosthodontists (professors or tenured lectured), but in the others the teaching was implemented by part-time prosthodontists. However, the practical teaching of CD is supervised by full-time prosthodontists in 63.2% of the schools, in the remaining 36.8% being supervised by part-time prosthodontists.

Q7: Material used for laboratory teaching.

The following results refer to dichotomous questions (Yes/No). Fourteen Schools (73.7%) reported using irreversible hydrocolloid as the preliminary impression material in edentulous Dentoform(TM) in manikin heads, and 63.2% reported making custom-trays, both self-cured and light-cured. All the schools surveyed teach students how to design the limits of acrylic record bases on the plaster model. Eleven schools (57.9%) reported using zinc oxide impression paste in custom trays for final impressions in preclinical teaching.

Q8: Mandibular positioning in the centric relation.

Eighteen schools (94.7%) used thumb and finger manipulation for positioning the mandible in the centric relation, but one school does not instruct students in jaw handling.

Q9: Articulator used and settings.

The following results refer to dichotomous questions (Yes/No). Eighteen schools (94.7%) reported using a semi-adjustable articulator in their clinical CD program, but only 15 schools (78.9%) use eccentric records to set the condylar trajectory and the Bennett angle of the articulator.

Q10: Tooth assembly.

The following results refer to dichotomous questions (Yes/No). Eighteen schools (94.7%) reported that their students make denture setups on wax. However the 73.7% of the surveyed schools instruct students to achieve a bilaterally balanced occlusion throughout occlusal adjustments and teeth-remounting. In this sense, 68.4% of the schools reported that their students are taught that customizing articulator parameters is essential for achieving a bilaterally balanced occlusion.

Q11: Preclinical assessment.

Fifteen schools (78.9%) perform a preclinical assessment of the competences achieved. Such assessments are only theoretical (31.3%), only practical (12.5%), or theoretical and practical (50.0%). Some criteria for such assessments have been explored in terms of relevance by schools that do carry out an examination, as follows. For example, the prosthetic prescription of the den-tures is considered to be important or very important by 76.9% of schools and to be unimportant according to the rest (23.1%). Record base retention is considered to be important by 92.9% of schools performing preclinical assessments. Moreover, the in-terocclusal records (vertical dimension, centric and eccentric relations) are also considered to be important by most schools (92.9%). The same proportion of schools considers the assembly of models on an articulator, the tooth set-up technique, and occlusal equilibration to be important.

-Clinical teaching

Q12: In which academic year does clinical teaching start?

Eleven schools (57.9%) start clinical training in the 4th or in the 5th year (36.8%). Only in one school do students start treating edentulous patients during their 3rd year (5.3%).

Q13: Student-instructor ratio in clinical sessions and activities.

The student-staff member ratio for clinical sessions was on average 10.6 ± 6.9 students, the range being between 5 and 30 students. In 14 schools (82.4%: a valid percentage since two schools did not respond), students work in pairs; in one school they work in threes, and in two schools 4 students work in the same box. The mean ratio for clinical activity was 2.3 ± 0.7 students/staff member.

Q14: Material used for final impressions.

Thirteen schools (68.4%) use custom trays instead of standard trays (31.6%) for making the final impression. Moreover, six schools (37.6%) use polyether as the primary material for final impressions and 5 responding schools use polyvinylsiloxane (31.3%) and another five schools (31.3%) mainly use alginates and/or zinc oxide pastes. Three schools (15.8%) did not respond to this item, and hence the previous percentages have been corrected accordingly.

Q15: Competence-based exam for CD.

Most of the 18 schools responding to this question (83.3%) reported that they do perform a competence-based assessment to see when the students are suitably prepared to treat CD patients, although most of them were still working on configuring a set of the most discriminative criteria for this assessment of competence.

Q16: Final examination.

Two schools did not respond to this item (10.5%). Of the other 17 schools, 41.2% gave a final exam comprising theoretical, preclinical and clinical sections. Four schools offered (23.5%) only a final written examination; three schools demanded both a pre-clinical and written final examination (17.6%); two schools carried out clinical and written examinations, and another school (5.9%) did not demand a final exam for CD.

Q17: Minimum CD requirements for graduation.

Eight schools (53.3%) indicated that students have ne-cessarily to make a minimum number of CDs in order to graduate, and 7 schools (46.7%) indicated that there was no such limit. On average, students constructed 1.8 ± 1.2 CD during their clinical training, ranging from 0 to 4. The distribution is shown in table 2. All the above values were estimated after correcting for the four non-responding schools (21.1%).

Q18: Clinical procedures for CD.

Thirteen of the 17 responding schools (76.5%) reported using a plastic compound as the primary material for border molding, whereas 4 (23.5%) declared no pattern in this customizing procedure. Among the same respondents, 9 schools (52.9%) reported that their students always performed a clinical remounting procedure and occlusal equilibration in the articulator before denture delivery, whereas 5 schools (29.4%) did so only in some cases, and 3 schools (17.6%) never.

Q19: Materials used for making CD.

Fifteen schools answered this item (78.9%) and indicated a percentage estimation of the materials used. On average, 88.7 ± 16.0 % of patients’ CD are made in acrylic, and the remaining 12.3 ± 15.7 %, are made in acrylic but with a metal reinforcement. Among these respondents (15 schools), 67.7% ± 44.0 % of the patients’ CD had anatomic teeth and 32.3% ± 42.4% had semi-anatomic teeth. Artificial teeth were mostly of acrylic (90.7% ± 25.8%) but also of ceramic in a lesser percentage (9.3% ± 25.8%).

Q20: Clinical Relining procedures.

Within the 17 responding schools, 9 (52.9%) indicated that their students are taught in relining denture bases but only using soft relining materials, and 3 (17.6%) with both hard and soft materials. However, in 5 schools (29.4%) the students never perform relining procedures.

Q21: Articulator used and settings for clinical teaching.

Within the 17 responding schools, five (29.4%) use Dentatus(TM) (Dentatus, Stockholm, Sweden); three (17.6%) use Stratos(TM) (Ivoclar Vivadent A.G, Schaän, Liechtestein); three (17.6%) use QuickMaster(TM) (FAG Dentaire, Cluses, France); two (11.8%) use WhipMix(TM)-8500 (Whip Mix Europe GmbH, Dortmund, Germany); two (11.8%) use Artex(TM) (Girrbach Dental GMBH, Pforzheim, Germany), and two (11.8%) use BioArt(TM)-4000 (Bio-Art LTDA. Sao Paulo, Brasil). However, only in 4 schools (23.5%) is the real axis of the condylar rotation located, and then only sometimes; the rest of the schools (76.5%) use an average axis.

Q22. Virtual video demonstrations.

Eighteen schools responded to this question (94.7%). Half of them declared that they had pre-recorded video demonstrations available for their students on the internet.

## Discussion

The results of this survey demonstrate the broad variety in the ways of the teaching of CD among Spanish and Portuguese universities before the implantation of the Bologna System, although several trends in the materials and procedures used are detected. The main coincidence is that all the schools surveyed teach the design of the record base on the plaster model. Also, most schools (89.5%) have a preclinical course of tooth mounting and denture design, mainly implemented during the third academic year of their respective courses (73.7%). This preclinical course is widely applied in both U.S. (6) and British Dental Schools (7), but in contrast to our results the majority of British Schools teach CD in the 4th and 5th years of their degree courses (7). Furthermore, our results demonstrate that the use of a manikin head for preliminary impressions in laboratory teaching is more widespread than in U.S. schools (6), although comparable to the situation in British schools (7).

In terms of quantitative teaching, the distribution of hours dedicated to lectures, laboratory work and clinical activities reveals that the teaching of CD is eminently a practical issue ( Table 1: Q3). The theoretical background is mainly conveyed through lectures or seminars, the use of innovative techniques, such as problem-based learning, and the use of online lectures ( Table 1: Q4) being exceptional, in agreement with Rashedi et al. (6), even though these techniques have proven effectiveness for students (8). However, it does appear that a virtual teaching is partially used for video demonstrations and uploading lectures ( Table 3: Q22).

According to Rashedi et al. (6), the overall mean of laboratory hours in preclinical courses among U.S. dental Schools is 74 hours, ranging from 31.5 to 160, which doubled the results of the present study. Castillo de Oyagüe et al. (9) reported that the duration of the preclinical course for removable partial dentures is clearly shorter in Spain, in comparison with U.S. and Great Britain. However, the mean number of lecture hours reported for U.S dental schools (6) (28 hours, ranging from 12 to 80) is similar to what was observed here. Our findings also seem to be in agreement with those reported by Sukotjo et al. (8) , referring to the preclinical hours spent on prosthetics implemented at the Harvard School of Dental Medicine.

Starting in 1970, some authors have documented the broad variations in student/professional staff ratios in Dental Schools (rang-ing from 1:3 to 1:33) (10). The student/staff ratio in clinical teaching found in the present survey ( Table 1: Q5) is similar to those reported for U.S. dental Schools (6,8), most of which have a ratio of 8:1 or higher, with an overall mean of 12:1. These results ( Table 1: Q5) are also fairly similar to those reported for British dental schools, ranging from 6 to 12 students per member of the professional staff (7). Nevertheless, these ratios are less than ideal, since the paired teaching (one student acting as the dentist and the partner as the assistant) reported by most of the schools surveyed ( Table 2: Q13) reduces the time available for individual students to gain hands-on experience. On the other hand, it is not convenient for a dentist to work alone, and hence a pair-based system could improve students’ team-working abilities. McGiveny (11) recommended a preclinical ratio of 1:10, and a clinical ratio ranging from 1:4 (first year) to 1:6 (for fixed and removable prostheses). Accordingly, the ratios reported here are slightly higher than this recommendation.

The percentage of full-time prosthodontists supervising CD teaching is similar to that reported by Rashedi et al. (6) for U.S. schools, and is perhaps one of the strengths of our traditional CD teaching. However, it is noticeable that full-time prosthodontists are mainly involved in theoretical teaching (84.2%) instead of in practical teaching (63.2%). This finding may be explained since in preclinical and clinical teaching several groups of student works simultaneously, each supervised by one member of the professional staff. However, in theory lectures there is only a single group, and this activity is mainly covered by full-time prosthodontists.

In terms of materials and procedures, most programs (95%) report using a semi-adjustable articulator and teaching jaw manipulation tests to establish the mandibular centric relation and eccentric records in order to customize the parameters of the articulator. The proportion of schools teaching a custom setting of semi-adjustable articulators instead of using average parameters is surprisingly high ( Table 1: Q9), as reported for the majority of U.S. schools (6). This information is only related to preclinical teaching, and hence future research should address the issue of whether this widely recommended procedure is also systematically applied in clinical teaching. The type of articulator used, as reported among U.S. schools (6), is heterogeneous, Dentatus(TM) (Dentatus, Stockholm, Sweden) being the most popular articulator trademark in Spanish and Portuguese dental schools ( Table 3: Q21).

For impressions, in consonance with previous research carried out in the U.S. (6,12) most dental schools in Spain and Portugal (73.7%) use alginate for primary impressions in dentoform. Conversely, the material used for final impressions is more heterogeneous, as reported by Clark et al. (7) for British schools, although there is a certain tendency to use silicones or polyether as the primary option, as reported Petrie et al. (13) for U.S. prosthodontists and dental schools. Nevertheless, in contrast to these authors (13), who reported a large proportion of prosthodontists using polysulfides for working impressions, among the Spanish and Portuguese schools surveyed polysulfide is not used.

Furthermore, most schools use custom trays for final impressions, although the percentage is lower than that reported among U.S. Schools (98%) (12). Most Spanish and Portuguese schools mold the border tray using plastic compounds, as reported elsewhere (13,14). Elastomeric impression materials have also been reported as alternative materials for border molding (13).

It is noteworthy that although the Bologna Declaration Agreement has only been introduced recently, and this system has not already affected to the subject of Prosthodontics, most schools do apply a uniform system of competence assessment, and most of them include an evaluation of clinical or preclinical skills in their final examination ( Table 2: Q16). Dentists in general and prosthodontists in particular, base their teaching on clinical practice, ( Table 1, Q3), affording newly graduated students a reasonably adequate professional training to enter the job market. However, this clinical evaluation has traditionally been based on the tutor’s continuous assessment of the gradual acquisition of clinical skills, and currently tutors have been called on (i.e., from the Bologna Accord) to standardize the minimum skills or procedural requirements to ensure the acquisition of professional competence. In this sense, we observed that there is no agreement about the minimum number of CD that students should make before graduation ( Table 2, Q17). Some reports indicate that most U.S schools establish a minimum of CD to be completed, but no official numbers have been published (12). In Great Britain, it has been suggested that students treating 6 or more edentulous patients would be adequately equipped for CD construction in vocational training (3). None of the schools surveyed reach this level, and thus authors consider that nowadays our students are not adequately skilled to treat edentulous patients by their own and without supervision. In addition, the mean number of CD constructed by each student per year in the present study (1.8 ± 1.2) seems to be insufficient to ensure adequate clinical skills. Clark et al. (3) pointed out this gradual decline in British dental schools. In any case, with this situation, recent Spanish and Portuguese graduates will be doomed to follow postgraduate courses to be instructed in this field, or to derive patients to trained prosthodontists.

Current evidence suggests that there will be a need for CD to be made in Spain (15) for decades. To our knowledge no epidemiological studies have been carried out in Portugal reporting data concerning edentulism in the elderly (16,17) but a similar pattern to that found in Spain would be expected. The data from the last national dental survey in Spain (15) revealed that the prevalence of CD wearers among the elderly has declined from 21.5% in 2000 to 15.6% in 2006. Moreover, the needs for CD in this latter survey were 4% and 5% in the upper and lower jaws of the elderly respectively. This need for CD will gradually decrease, but it is foreseen that, in general, cases will become more difficult since the supporting oral tissues and tolerance to dentures are reduced with aging (18).

The present study highlights the main descriptors of the teaching of CD in Spain and Portugal, although this is only based on the perceptions of instructors. Thus, there is clearly a need to contrast this information by incorporating the views of trainees and this should be addressed in future studies.

## References

[1] Sharry J (1970). Prospects for prosthodontics. J Prosthet Dent.

[2] Lang BR (1994). A review of traditional therapies in complete dentures. J Prosthet Dent.

[3] Clark RK (2002). The future of teaching of complete denture construction to undergraduates. Br Dent J.

[4] Bravo M (2002). Private dental visits per dentist in Spain from 1987 to 1997. An analysis from the Spanish national Health Interview Surveys. Community Dent Oral Epidemiol.

[5] Graser GN (1990). Predoctoral removable prosthodontics education. J Prosthet Dent.

[6] Rashedi B, Petropoulos VC (2003). Preclinical complete dentures curriculum survey. J Prosthodont.

[7] Clark RK, Radford DR, Juszczyk AS (2010). Current trends in complete denture teaching in British dental schools. Br Dent J.

[8] Sukotjo C, Thammasitboon K, Howell H, Karimbux N (2008). Students' perceptions of prosthodontics in a PBL hybrid curriculum. J Prosthodont.

[9] Castillo de Oyagüe R, Lynch C (2011). Variations in teaching of removable partial dentures in Spanish dental schools. Med Oral Patol Oral Cir Bucal.

[10] Levin B, Sauer JL Jr (1970). Survey of complete denture procedures taught in thirty-three dental schools in the United States and Canada. J Dent Educ.

[11] McGivney GP (1976). Prosthetic teachers: number and qualifications for undergraduate education. J Prosthet Dent.

[12] Petropoulos VC, Rashedi B (2003). Current concepts and techniques in complete denture final impression procedures. J Prosthodont.

[13] Petrie CS, Walker MP, Williams K (2005). A survey of U.S. prosthodontists and dental schools on the current materials and methods for final impressions for complete denture prosthodontics. J Prosthodont.

[14] Jaggers JH, Javid NS, Colaizzi FA (1985). Complete denture curriculum survey of dental schools in the United States. J Prosthet Dent.

[15] Bravo M, Cortés J, Casals E, Llena C, Almerich-Silla JM, Cuenca E (2009). Basic oral health goals for Spain 2015/2020. Int Dent J.

[16] de Almeida CM, Emílio MC, Moller I, Marthaler T (1990). 1st exploratory national survey of disease prevalence and treatment needs of the oral cavity. Rev Port Estomatol Cir Maxilofac.

[17] Davis RK, Meyer K, Freitas E, Kristoffersen T (1983). Tooth loss and prosthetic replacement in Portugal. A baseline study in youths and adults. Rev Port Estomatol Cir Maxilofac.

[18] Lechner SK (1982). Overcoming the adaptational problems with complete and partial dentures. Int Dent J.

